# Hygiene Assessment of Buffalo Milking Parlours in Campania Region, Italy: A Preliminary Study by Using ATP Luminometry and Bacteriological Investigation

**DOI:** 10.3390/ani14121805

**Published:** 2024-06-17

**Authors:** Valentina Iovane, Andrea Fulgione, Francesca Pizzano, Angelo Masullo, Emine Ipek, Giuseppe Parente, Francesca Paola Nocera, Luisa De Martino

**Affiliations:** 1 Department of Agricultural Sciences, University of Naples ‘Federico II’, Via Università 100, 80055 Portici, Italy; 2 Department of Veterinary Medicine and Animal Production, University of Naples ‘Federico II’, Via F. Delpino 1, 80137 Naples, Italy; 3 ASL Salerno, Via Nizza 146, 84122 Salerno, Italy; 4 Centro di Riferimento Regionale per la Sanità Animale (CRESAN)—Dipartimento di Prevenzione, Corso Garibaldi 5, 84122 Salerno, Italy; 5 Task Force on Microbiome Studies, University of Naples ‘Federico II’, 80137 Naples, Italy

**Keywords:** ATP luminometry, buffalo farm, milking parlour surfaces, bacteria isolation

## Abstract

**Simple Summary:**

In dairy buffalo farms, the hygiene of the milking area represents a key task of internal biosecurity measures since it can directly influence the dairy animals’ health and the microbiological quality of raw milk. The aim of this study was to evaluate the cleanliness and the bacterial contamination of milking parlours’ surfaces and equipment of eight buffalo farms located in the Campania Region using an ATP-bioluminescence assay and bacteriological analysis during the year 2022. The findings of this study underline that an ATP-bioluminescence assay is a valid, complementary, cost-effective on-farm tool useful to quickly detect the contamination of milking parlours.

**Abstract:**

Careful cleaning of a milking parlour and its equipment is fundamental to guarantee good raw milk quality and prevent the dissemination of bacteria and improve animal welfare. This study aimed to investigate, using an ATP-bioluminescence assay and bacteriological analysis, the bacterial contamination of milking parlours on milking parlour surfaces of buffalo farms in the Campania Region, evaluating the seasonal dynamics during the year 2022. Eight farms were selected by the Italian ClassyFarm system, which assesses the level of animal welfare and biosecurity according to risk analysis. Before sampling, all dairy farm owners filled out a questionnaire on milking management, animal hygiene, and health. The questionnaires evidenced similar cleaning procedures but an absence of a standardised cleaning protocol among the different farms. ATP bioluminescence results evidenced similar levels of contamination in all the selected buffalo farms, and the season comparison showed no significant differences. A variation in the percentages of bacterial isolates during the different seasons was observed, with a higher prevalence of *Enterobacteriaceae* (38%) in summer. A small number of samples exhibited an absence of bacterial growth. Identifying bacteria is crucial for understanding the microorganisms present in the milking parlour, yet employing ATP luminometry could offer broad and accurate applications in buffalo milking parlours. In conclusion, the use of ATP bioluminescence for evaluating the hygiene of a buffalo milking parlour could represent a further important advancement in dairy farming technology.

## 1. Introduction

The cleanliness of the milking parlour and milking equipment represent a relevant aspect of the dairy industry since it can directly affect the bacteriological quality of raw milk, milking process performances, and also the dairy animals’ health [1,2]. Indeed, to control diseases and improve animal welfare, dairy farms have adopted standard cleaning practices to avoid the spread of opportunistic and/or pathogenic bacteria from one animal to the next during milking shifts [2,3]. Despite this, bacterial contamination of the milking parlour surface and equipment still represents a critical point in the management of dairy farms, as the effectiveness of cleaning procedures can be influenced by several factors such as water quantity and temperature, the type and concentration of the detergent, and equipment maintenance [4]. Thus, bacterial colonization can accumulate, especially on milking equipment, when the cleaning procedure is not functioning properly, leading often to biofilm formation, which should be prevented to ensure good hygiene quality of raw milk [4,5]. 

Several methods are available to evaluate the hygiene of the milking parlour area and its equipment. Currently, visual inspection is the most commonly used verification practice for its ease and speed of execution on farms. However, this practice has many limitations such as the reduced sensitivity in detecting the dirt if the equipment and surfaces are not extremely contaminated and, obviously, the lack of objectivity, as its outcomes mainly depend on the operator’s assessments [6,7]. Classic bacteriological culture analysis of surface swabs is considered the “gold standard” in detecting the bacterial contamination of the equipment and environment, being a scientifically validated and objective method. However, the results of laboratory culture-based testing are not immediately available, making the rapid and timely assessment of cleanliness difficult, and consequently delaying the on-farm cleaning practices [4,6]. Thus, ATP bioluminescence represents a good complement when it is important to obtain results quickly [8]. In light of the drawbacks of the previously described methods, ATP bioluminescence technology has been implemented in the dairy industry and in swine farms in recent years [2,3,6,9,10,11], since the rapid assessment of ATP on surfaces and equipment has been described as an indirect, rapid, and easy-to-use on-farm tool to detect and quantify the bacterial contamination and to check the effectiveness of adopted cleaning practices [12].

The ATP bioluminescence technique has evolved during the last decades, and it has become increasingly used, mainly in healthcare settings to evaluate the hygiene of various hospital surfaces and reusable surgical instruments [7,13,14] and in the food industry [15,16]. The presence of ATP on surfaces indicates improper cleaning and the presence of contamination, including organic debris and bacteria. Indeed, the method is based on a reaction between ATP molecules and the enzyme luciferase with its substrate luciferin [17]. In this reaction, ATP is converted to AMP with the emission of light at an intensity that is directly proportional to the amount of ATP in the sample [18]. Therefore, this technique indirectly measures the number of microorganisms in a sample, giving results in a few minutes. This is the reason why ATP bioluminescence can be effectively used under field conditions, but it is recommended to combine it with microbiological testing because other nonbacterial organic materials can also contribute to ATP measurement [18,19,20].

From the literature, it is known that ATP bioluminescence has previously been assessed to investigate only the hygienic status of milking equipment in dairy farms, but here it is reported for the first time its use in the buffalo milking parlour environment. In this study, specific surfaces of the milking parlour of eight different buffalo farms were swabbed both with ATP surface swabs and transport swabs for bacteriological testing during the four seasons of 2022.

## 2. Materials and Methods

### 2.1. Ethical Statement

This study was approved by the Institutional Animal Ethics Committee of the University of Naples Federico II (Italy) PG/2023/0077653_29/06/2023. All the samples of this study were from different buffalo farms and were taken during routine evening milking shifts. Milking parlour surface samples were collected with the farm owner’s permission, and no written informed consent was required. No animals were used in this study. 

### 2.2. Participant Farms and Questionnaire Survey

In this study, eight buffalo farms located in the Campania Region, precisely in the Salerno Province, were selected. In particular, farms with a number of animals not exceeding 500 were chosen according to the ClassyFarm system using the options of similar risk indexes relating to biosecurity, animal welfare, health (both animal and public health), production data, nutrition, and drug consumption.

**Figure 1:**
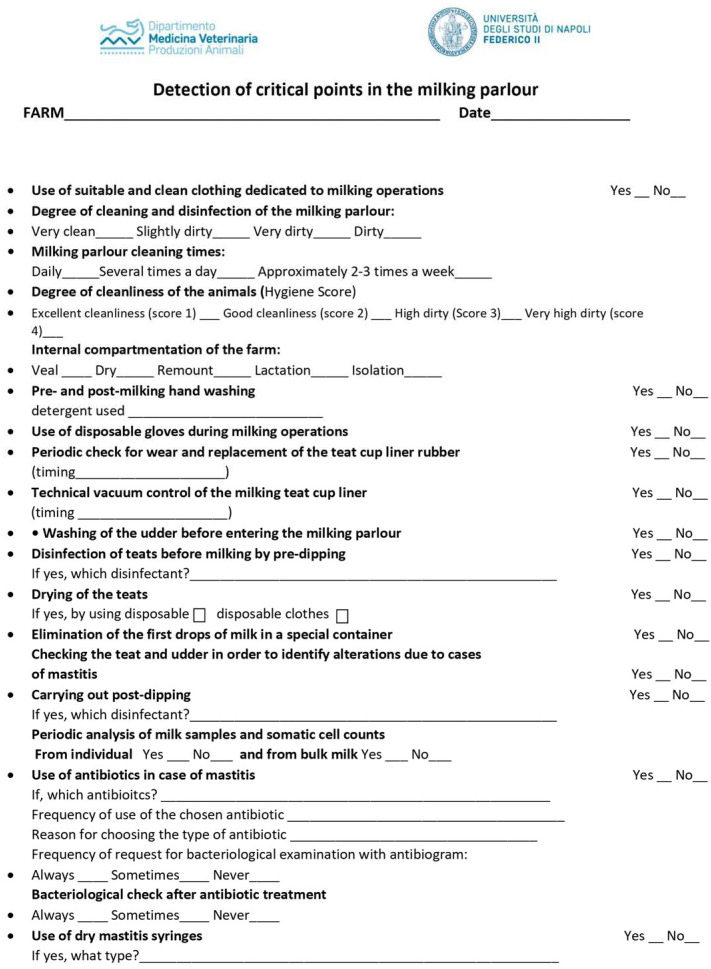
Farm questionnaire survey.

Each farm was sampled 4 times during the evening milking shift of the different seasons. Firstly, dairy farm owners were requested to complete a questionnaire at the beginning of the study period, to detect and collect information on milking management, hygiene, and animal health to evaluate any critical points of the “milking system” (Figure 1).

The survey consisted of a series of 19 questions having mainly a yes-no choice format, with the possibility to provide additional information. The questions focused on the cleaning practices performed before and after the milking shifts. Moreover, the degree of cleanliness of the animals was evaluated by the trained operator (author V.I. of the manuscript), taking into account a 4-category scale: (1) total cleanliness of udder, flank/upper legs, and lower legs; (2) dirty only flank/upper legs or lower legs; (3) dirty in two of the three considered body sites; (4) very high dirtiness of udder, flank/upper legs and lower legs.

### 2.3. Collection of Samples 

In the milking parlour of each selected farm, well-defined areas of the milking room and milking equipment surfaces were sampled using ATP Ultrasnap surface swabs (Hygiena LLC, Camarillo, CA, USA) and bacterial transport swabs (Aptaca Spa, Asti, Italy). The sampling was performed by the same trained operator (author V.I.), who collected the ATP swabs according to the manufacturer’s instructions (SystemSurePlus Luminometer, Hygiena, UK). Sampled areas included the inner surface of the teat cup liner, the surfaces of the wall of the external animal passage, and the floor of the milking zone. Precisely, each sample consisted of a 10 × 10 cm area and the sampling was performed by rotating the swab 360 degrees to ensure optimal contact with the area to be tested. 

### 2.4. ATP Bioluminescence Assay 

The ATP bioluminescence test was performed on the farm using the Bioluminometer SystemSure Plus (Hygiena LLC, Camarillo, CA, USA) with ATP Ultrasnap Surface swabs (Hygiena LLC, Camarillo, CA, USA) according to the manufacturer’s instructions. The results were reported as relative luminescence units (RLU). Once sampling took place, the swab was placed in its protective cap, the capsule contained in the swab cap was broken by the pressure of the fingers, and the luciferase reagent was brought into contact with the sample. Once the reaction was activated, the swab was inserted into the luminometer within 60 s and the result was displayed approximately after 15 s. The luminometer displayed a value expressed in RLU which was directly proportional to the concentration of ATP present in the sample. For each collected swab, the RLU was read twice consecutively, and the average value was then calculated.

Murphy et al. [21] set the threshold for a “clean surface” at ≤100 RLU, between 100 and 150 RLU for surfaces with deficient cleanliness, and a value above 150 RLU for dirty or very deficient surfaces. Most recent publications, performed in cattle farms indicated a higher value for a “dirty surface”, precisely above 300 RLU [4] or above 1000 RLU [11]. Considering the behavioural differences and the different housing conditions between bovine and buffalo breeding, for instance, especially in the summertime, the Mediterranean buffalo’s habit of wallowing in mudholes to cool off compared to cattle which prefer pasture grazing in dry environments, in this study a value of >1000 RLU was adopted for a non-compliant degree of cleaning.

### 2.5. Bacterial Isolation and Identification

Once delivered to the laboratory within 24 h of the sampling, qualitative microbiological tests were carried out with standard protocols on the swabs collected from the milking parlour environment. The swabs were streaked on a solid culture media for bacterial isolation. Mannitol Salt Agar (MSA) was used to selectively isolate Staphylococci, Mac-Conkey Agar (MCA) was used to selectively isolate Gram-negative bacteria, and Columbia CNA agar was used to isolate Gram-positive bacteria. All the plates were purchased from Liofilchem Srl (Teramo, Italy).

After the incubation of the culture media at 37 °C for 24 h, the recovered colonies were first evaluated for their morphologic features on agar plates, and then the bacterial identification was performed by using matrix-assisted laser desorption/ionization–time of flight mass spectrometry (MALDI–TOF MS) (Bruker Daltonics Inc., Bremen, Germany), according to manufacturer’s guidelines. A bacterial test standard (BTS) (Bruker Daltonics, Bremen, Germany) was used as a calibrator for quality control.

### 2.6. Statistical Analysis

Descriptive statistical analysis was employed to evaluate the values of the degree of hygiene obtained through the bioluminometry sampling. All graphics were made using Excel software. The standard deviation (SD) of the mean was additionally calculated. All generated results by the Microbiological Diagnostic Laboratory were recorded and entered into a Microsoft 365 Excel™ spreadsheet for successive analysis.

Luminometry results of the sampling areas, obtained for each season, were analyzed using a One-Way ANOVA Calculator, including Turkey HSD (Statistical Program Easy Fisher Exact Test Calculator) to determine if there was a significant difference in relation to the sampling season.

## 3. Results

### 3.1. Farm Questionnaire Survey

A total of eight questionnaires were collected, and, precisely, the questionnaire was administered to each farm before the first sampling. All data collection was performed by the same trained operator (author V.I.) who documented by visual assessment that almost all farms exhibited a slightly dirty milking parlour with a worsening during the summer period.

The results showed that all the farms selected for this study had similar cleaning procedures. In particular, all the farms adopted suitable and clean disposable clothing for milking operations and carried out the milking parlour cleaning operation at the end of the workday (i.e., after the evening milking shift). Instead, differences were documented by the milking parlour staff between pre- and post-milking hand washing, such as the type of detergent used (hand-wash paste or liquid hand soap or only tap water).

The answers obtained about the periodic check for wear and replacement of the rubber of the teat cup liners varied among the selected farms. This operation was carried out every six months by five farms while the remaining ones performed it every two months; however, in the case of sheath malfunction, they were always immediately changed with new ones. 

Checking the teat to identify alterations due to cases of mastitis was carried out at every milking on all farms; the analysis of milk samples and somatic cell counts from bulk milk was performed twice a month.

Referring to antibiotic administration in the case of clinical mastitis, it occurred only after farm veterinary prescription, with ampicillin or cefalexin reported as the most used molecules. However, the survey highlighted the absence of the bacteriological examination request with an antibiogram, thus displaying the lack of this well-known and useful diagnostic procedure.

### 3.2. ATP Bioluminescence Assay Results

A total of 96 ATP swabs were collected at each selected milking parlour surface. Specific zones of the milking parlour were sampled: the inner surface of the teat cup liner, the wall of external animal passage in the milking room, and the floor of the milking zone during the schedule of sampling (4 seasons).

**Figure 2:**
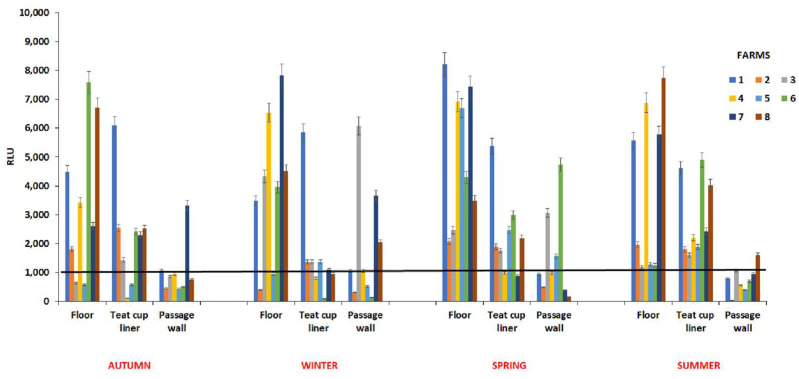
ATP bioluminescence assay measurements.

The results showed great variability during the different seasons both among the selected farms and for the sampled areas (Figure 2). In particular, the value of bioluminescence measurement obtained from passage wall samples of almost all the farms was lower or equal to 1000 RLU (chosen RLU threshold of acceptable cleaning), overall, in summer and autumn. Precisely, the passage wall samples showed in autumn (farm n. 7) and in summer (farm n. 8) RLU results higher than the established threshold, 3500 and 1500 RLU, respectively. With regards to the inner surface of the teat cup liner, farms n. 4 and 5 in autumn, farms n. 4, 6, 7 and 8 in winter, and farms n. 4 and 7 in spring showed values lower than 1000 RLU; whereas in summer the recorded values were higher than 1000 RLU in all the farms. Particularly, the farm n. 1 showed very high values, around 6000 RLU, in autumn, winter, and spring, with a slight decrease in summer, around 4500 RLU (Figure 2). In winter, three farms (n. 2, 3 and 5) displayed values never higher than 1500 RLU. For farms n. 6 and 8, a relevant increase was recorded (>4000 RLU) in the summer. The highest RLU values were found on the floor of all the farms in spring, with 8000 RLU in farm n. 1, probably due to the fact that the sampling was carried out on rainy days; however, also in summer for farms n. 1, 4, 7, and 8, RLUs > 6000 were measured, likely related to buffaloes’ habit of wallowing in mudholes to cool off. However, the farm identified as n. 5 (Figure 2) turned out to be the cleanest one, exhibiting low RLU (<1000) values in autumn, winter, and spring. All data derived from RLU measurements were analysed using the pairwise comparison procedure between the different seasons, and no significant results with *p* < 0.05 were obtained. 

### 3.3. Identification of Bacteria Isolated from Milking Room Surfaces

**Figure 3:**
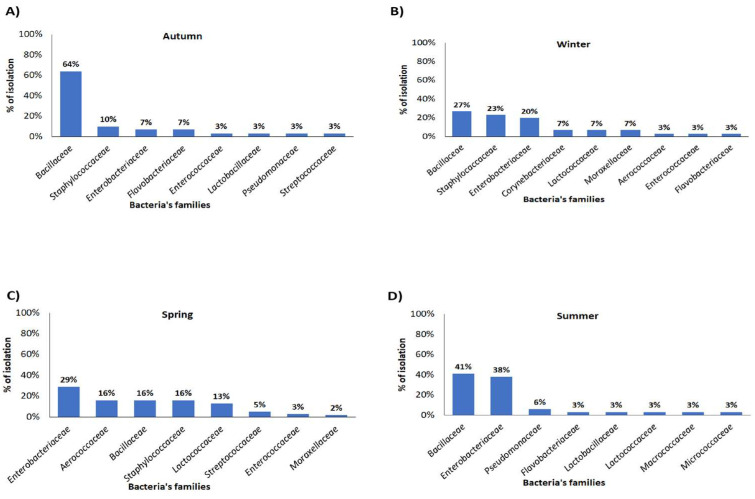
Identification and seasonal variation of bacteria isolated from milking parlour equipment in 2022: (**A**) Autumn bacteriological sampling; (**B**) Winter bacteriological sampling; (**C**) Spring bacteriological sampling; (**D**) Summer bacteriological sampling.

A total of 96 transport swabs were collected from the milking parlour surfaces, which were also sampled with ATP swabs. Bacterial isolation and identification results evidenced a variation in the percentages of bacterial isolates during the different seasons (Figure 3). No bacterial growth was detected in some samples, with 3/24, 2/24, 4/24, and 4/24 in autumn, winter, spring, and summer, respectively. 

As reported in Table 1, a limited quantity of samples displayed no bacterial growth. Low isolation percentages (3% < x < 7%) were recorded for the other bacteria families as shown in Figure 3.

Among positive samples, a higher prevalence of *Bacillaceae* was found during autumn and summer with a rate of 64% and 41%, respectively (Figure 3C,D), while during colder months the isolation frequency was below 30% (Figure 3A,B). No *Staphylococcaceae* was isolated in the summer sampling, while isolation values of 10%, 16%, and 23% were recorded in autumn, spring, and winter, respectively. Conversely, an increased frequency of isolation was observed for *Enterobacteriaceae* during the warmer months, with levels of 29% in spring and 38% in summer (Figure 3C,D).

As shown in Table 2, during the spring sampling, *Enterobacteriaceae* were detected in the floor (3/8; 37.5%), the inner surface of the teat cup liner (4/8; 50%) and the passage wall (3/8; 37.5%) swabs; however, in summer, *Enterobacteriaceae* were identified from the swabs of both the inner surface of the teat cup liner (6/8; 75%) and the floor (5/8; 62.5%) in almost all the selected farms. Furthermore, general hygiene/sanitary conditions in the environment were not up to standard in farm n. 4, since 6/12 (50%) samples were positive for *Enterobacteriaceae* growth, especially in winter and in spring. Further, farm n. 3 which presented 5/12 (41.7%) samples positive to *Enterobacteriaceae* growth, displayed spring season positivity in all three kinds of samples (floor, inner surface of the teat cup liner, and passage wall) (Table 2).

## 4. Discussion

In 2018, the Italian Ministry of Health introduced the integrated system ClassyFarm (https://www.classyfarm.it) [22] for categorizing the risk level of the farms, giving particular attention to animal welfare and biosecurity. This platform can be consulted by veterinarians for monitoring, analysing, and performing interventions according to the European Regulation (UE) N. 2016/429 of 09 March 2016 [23]. This provides an opportunity for all farmers to enhance their practices, aiming for excellence. The fundamental pillars of ClassyFarm are animal welfare, pharmacosurveillance, and biosecurity. The collection of information relating to self-control activities is carried out by farm operators and surveillance activities are performed by official veterinarians. 

For this study, the Campania Region ClassyFarm system used to select the farms, showing similar parameters according to risk analysis, was employed. The results of the questionnaires submitted to farm owners evidenced similar standard cleaning operating procedures adopted in each farm for the milking parlour management. In particular, the cleaning practices of the milking parlour were performed by the farm workers at the end of the evening milking shift in all selected farms. 

The milking parlour is a high-density place used several times a day, making regular cleaning imperative to prevent pathogen proliferation in this environment. Just as the milking machine undergoes daily cleaning, so too should the milking parlour itself. Following each milking shift, it is essential to rinse the parlour thoroughly with water, and generally, a weekly regimen of detergent cleaning followed by disinfection is recommended to maintain optimal hygiene standards.

A previous article by Stefan and Baraitareanu [24] suggested that to prevent pathogen proliferation in dairy farms, milking parlour surfaces should be cleaned and disinfected twice daily. 

Furthermore, here it was documented that all farm workers wore suitable personal protective equipment (milkers’ gloves and milkers’ overalls) during the milking operations, even though all of them preferred to wear reusable work rubber gloves instead of disposable ones; the latter was interpreted by us as a critical point of hygiene management. It is well-known that proper hygiene procedures reduce microorganisms, somatic cells, and mastitis frequency, improving milk quality and cow health [25].

However, it is worth noting that during milking procedures, buffaloes’ teats were also checked to find possible alterations, such as clinical mastitis signs. Of course, subclinical mastitis may be missed in this kind of check. Subclinical mastitis, on the other hand, has no visible symptoms and can only be diagnosed with laboratory methods which depend on two parameters, the microbiological profile of sampled milk and the somatic cell count [26,27]. 

In any case, the majority of farms involved in this study complied with the recommendations given at the first visit for adequate milking practices, which did not necessarily imply compliance with a consistent timing protocol. Determining whether or not cleaning protocols are followed on the farm can be a key area to focus on to improve milk quality and also to control mastitis. If producers could be motivated to change and were trained to implement a correct milking preparation routine, improvements in these practices could be experienced and maintained. Moreover, differences were observed for hand washing before and after milking among the staff of the different farms, with five of the interviewed dairy farm owners reporting the use of detergent (liquid hand soap and hands-wash paste), while three of them declared the use of only tap water. A greater variability was observed for the milking parlour equipment relating to the times of the technical vacuum checking of the milking machine and the replacement of the rubber of teat cup liners, indicating a lack of standardization in the equipment management. Therefore, the collected answers revealed that cleaning procedures were not properly performed. In this regard, it is known that maintaining adequate hygiene practices in milking parlours is crucial for reducing bacterial exposure and dissemination, and consequently improving animals’ health and welfare [28]. 

Referring to the ATP bioluminescence assay, a threshold of >1000 RLU was herein adopted, as described by Buczinski et al. [10] for the cleanliness evaluation of the surfaces of dairy farms. During the sampling period, the detected RLU values showed a variation among the different seasons. Precisely, the highest RLU values were found on the floor of all the farms, especially on rainy days during the spring sampling, and in summer when the buffaloes wallow in mudholes. Indeed, it is known that the Mediterranean buffalo is a shade and water-loving animal, different from cattle that prefer pasture grazing in dry environments. Buffaloes are well-adapted to hot and humid climates as well as muddy terrain due to their unique morphological, anatomical, and behavioural traits. 

The accurate and definitive identification of bacteria is one of the cornerstones on which the fields of microbiology and infectious diseases are jointly based. Therefore, it remains fundamental to understand which microorganisms circulate in the milking parlour. 

This study showed that several potential sources of variation were mainly due to different seasonality. In fact, our findings demonstrated fluctuating percentages of bacterial isolates across distinct seasons. It was intriguing to note a higher incidence of *Enterobacteriaceae* isolation during the warmer months, particularly in summer, when 38% of the strains were isolated, and this positivity was found for the inner surface of the teat cup liner swabs in six out of the eight selected farms. This latter finding is really worrying because, as it is well known, bacteria belonging to the *Enterobacteriaceae* family play a significant role as the primary cause of dairy mastitis, a condition with severe repercussions on the health of dairy animals and the production of milk [29]. It has also already been reported that poor hygienic conditions can be associated with a high presence of *Enterobacteriaceae* in raw milk during summer [30]. *Enterobacteriaceae*, known for their pathogenicity, play a significant role in various aspects, particularly in food safety. Commonly linked to intestinal infections, they are ubiquitous in natural environments, and overall, they are considered as indicators of sanitary practices during milking for microbial quality and hygiene [31].

The bacteriological testing results partially corresponded to the RLU measurements, because for each season about three samples did not give bacterial growth even if the RLU values resulted to be high. This partial correspondence between the ATP bioluminescence assay and bacteriological analysis was due to the fact that the ATP luminometry technique quantifies not only ATP from bacteria but also from eukaryote cells, parasites, and organic materials such as milk residues in milking parlour environments [32]. Moreover, it is important to point out that several factors, such as the sampling location and type of detergent used, influence the ATP levels, thus making its measurement reproducibility difficult [4].

Despite these limitations, an ATP bioluminescence assay may be considered a valid, complementary, cost-effective, on-farm tool useful to quickly detect contamination, above all when it is not directly apparent at visual inspection [3,11]. Therefore, an ATP bioluminescence assay gives the possibility to swiftly assess the cleanliness of the environment and supplied equipment on the field, but it cannot substitute laboratory culture-based testing, which allows it to really reveal the bacterial contamination of surfaces [20]. As reported by Chancy et al. [11], to obtain a clear and complete image of the hygiene of a farm, the ATP bioluminescence assay and bacteriological analysis are two techniques which should be applied together to collect different but complementary information about the hygienic status of a farm. Regardless of its observational feature, this study obtained interesting preliminary insights on the usefulness of ATP luminometry in the routine management of buffalo milking parlours. However, these findings propose that integrating on-farm ATP testing with visual inspection presents a feasible approach for evaluating cleanliness levels; not so much for the surfaces such as the walls and floor of a milking parlour, but, above all, for the equipment of the milking parlour.

Although not intended to replace culture methods, the ATP bioluminescence test is a valuable tool for evaluating the effectiveness of cleaning protocols. By offering timely feedback, the ATP test improves operator awareness and facilitates rapid intervention in critical circumstances.

## 5. Conclusions

Livestock biosecurity mitigates the risk of pathogens infiltrating and proliferating within livestock facilities by implementing measures like thorough sanitation and disinfection protocols. These findings suggest that using on-farm ATP testing in conjunction with visual inspection may serve as a practicable method to assess cleanliness levels, offering an on-farm complementary tool to traditional laboratory culture-based techniques for the detection and identification of the various microbial species present. The ATP bioluminescence test represents a notable advancement in agricultural technology, providing a rapid and effective means of assessing the health and condition of the buffalo milking parlour, consequently and indirectly safeguarding the quality of buffalo milk. Further research may be useful to introduce this method into the adopted procedures from the ClassyFarm system.

## Figures and Tables

**Table 1:** Absence of bacterial growth in the listed samples from buffalo milking parlours with the relative RLU values.

Farm ID	Sample Type	Season	RLU ± SD
6	floor	autumn	7589 ± 5.65
7	teat cup liner inner surface	autumn	2291 ± 1.41
8	floor	autumn	6710 ± 42.42
5	passage wall	winter	514 ± 5.65
6	floor	winter	3952 ± 2.82
1	passage wall	spring	940 ± 5.65
2	passage wall	spring	483 ± 4.24
7	floor	spring	7434 ± 21.21
7	passage wall	spring	381 ± 12.72
2	passage wall	summer	45 ± 7.07
4	passage wall	summer	559 ± 4.24
5	passage wall	summer	407 ± 8.48
6	passage wall	summer	702 ± 2.64

**Table 2:** *Enterobacteriaceae* identification from various samples of the milking parlours.

	Autumn			Winter			Spring			Summer		
Farm ID	A *	B **	C ***	A	B	C	A	B	C	A	B	C
1	-	-	-	-	-	-	-	-	-	X	X	-
2	-	X	-	-	-	-	X	X	-	X	-	-
3	-	-	-	-	-	X	X	X	X	-	X	-
4	-	-	-	X	-	X	X	X	X	-	X	-
5	-	-	-	-	X	-	-	-	X	-	X	-
6	-	-	-	-	-	-	-	-	-	X	X	-
7	-	-	-	-	-	-	-	X	-	X	-	-
8	-	-	-	X	-	-	-	-	-	X	X	-

* A = Floor; ** B = inner surface of teat cup liner; *** C = Passage wall; X = *Enterobacteriaceae* detection.

## Data Availability

The data represents the findings of this study, and they are available within the article.
